# Supplementary material for: A Critical View of the Application of the APEX Software (Aqueous Photochemistry of Environmentally-Occurring Xenobiotics) to Predict Photoreaction Kinetics in Surface Freshwaters
Source: Molecules. 2019 Dec 18;25(1):9. doi: 10.3390/molecules25010009 (PMC7017383; doi:10.3390/molecules25010009)
Supplement: Supplementary file 1 [file molecules-25-00009-s001.zip › 3.2.4_gcc-4.4.0.zip!/3.2.4_gcc-4.4.0/3.2.4_gcc-4.4.0/doc/octave/html/liboctave/Nonlinear-Programming.html]

Nonlinear Programming - Octave C++ Classes


Previous: Quadratic Programming,
Up: Optimization


---

### 9.6 Nonlinear Programming

— : **NLP** (void)  
— : **NLP** (const ColumnVector &x, const Objective &phi)  
— : **NLP** (const ColumnVector &x, const Objective &phi, const Bounds &b)  
— : **NLP** (const ColumnVector &x, const Objective &phi, const Bounds &b, const LinConst &lc)  
— : **NLP** (const ColumnVector &x, const Objective &phi, const Bounds &b, const LinConst &lc, const NLConst &nlc)  
— : **NLP** (const ColumnVector &x, const Objective &phi, const LinConst &lc)  
— : **NLP** (const ColumnVector &x, const Objective &phi, const LinConst &lc, const NLConst &nlc)  
— : **NLP** (const ColumnVector &x, const Objective &phi, const NLConst &nlc)  
— : **NLP** (const ColumnVector &x, const Objective &phi, const Bounds &b, const NLConst &nlc)

— : NLP& **operator =** (const NLP &a)

— : int **size** (void) const

— : ColumnVector **minimize** (void)  
— : ColumnVector **minimize** (double &objf)  
— : ColumnVector **minimize** (double &objf, int &inform)  
— : ColumnVector **minimize** (double &objf, int &inform, ColumnVector &lambda)

— : ColumnVector **minimize** (const ColumnVector &x)  
— : ColumnVector **minimize** (const ColumnVector &x, double &objf)  
— : ColumnVector **minimize** (const ColumnVector &x, double &objf, int &inform)  
— : ColumnVector **minimize** (const ColumnVector &x, double &objf, int &inform, ColumnVector &lambda)
